# Assessing the impact of an online dementia awareness initiative co‐created with and for English, Arabic and Vietnamese speaking communities: A case study

**DOI:** 10.1111/hex.14026

**Published:** 2024-04-15

**Authors:** Yousra Ali, Gabriela E. Caballero, Eman Shatnawi, Ann Dadich, Genevieve Z. Steiner‐Lim, Canterbury Bankstown Dementia Alliance, Michelle DiGiacomo, Diana Karamacoska

**Affiliations:** ^1^ School of Psychology Western Sydney University Penrith Australia; ^2^ NICM Health Research Institute Western Sydney University Penrith Australia; ^3^ Translational Health Research Institute (THRI) Western Sydney University Penrith Australia; ^4^ School of Business Western Sydney University Penrith Australia; ^5^ City of Canterbury Bankstown Australia; ^6^ Improving Palliative, Aged and Chronic Care through Clinical Research and Translation (IMPACCT), Faculty of Health University of Technology Sydney Broadway Australia

**Keywords:** Alzheimer's disease, dementia, education, knowledge, multicultural, multilingual

## Abstract

**Background:**

Awareness and understanding of dementia remain limited in ethnically diverse populations in multicultural societies due to culturally inappropriate and inaccessible information.

**Objective:**

To establish the impact, helpers and hinderers of an online multilingual dementia awareness initiative co‐created with and for English, Arabic and Vietnamese speaking people.

**Design:**

A case study using mixed methods to assess the impact and implementation of an information session on dementia knowledge.

**Setting and Participants:**

The study was conducted with English, Arabic and Vietnamese speaking individuals in Canterbury‐Bankstown, Australia.

**Intervention Studied:**

A dementia alliance co‐created an online multilingual dementia information session, which was delivered synchronously in English, Arabic and Vietnamese by trained facilitators.

**Main Outcome Measures:**

In‐session group discussions, quizzes and a postsession survey assessed the impact on dementia knowledge. A postimplementation focus group explored the factors that helped and hindered the initiative.

**Results:**

The online dementia information session successfully supported participants understanding of dementia causes, impacts and care strategies. The initiative was hindered by competing priorities and limited accessibility to target audiences, while it was helped by the support of an established organisation and feedback mechanisms.

**Discussion:**

Ongoing dementia education and awareness‐raising campaigns that are culturally sensitive are needed in communities to promote dementia literacy and help‐seeking.

**Conclusions:**

An online multilingual dementia information session can be an effective way to improve dementia literacy and advocate for change in multicultural communities.

**Patient or Public Contribution:**

English, Arabic and Vietnamese speaking members of the Canterbury Bankstown Dementia Alliance participated in the co‐creation and evaluation of this initiative.

## 1 INTRODUCTION

Dementia is a syndrome characterised by cognitive and functional decline beyond the scope of normal biological ageing processes.^1^ Alzheimer's disease and related dementias are the seventh leading cause of death around the world.^2^ However, in Australia, where this study is set, it is the second leading cause of death (the leading cause in women) and the leading cause of disability in people aged 65 years and over.^3^ Despite growing prevalence rates, limited awareness and understanding of dementia remain, resulting in negative and discriminatory beliefs.^4^ Stigmatisation contributes to poorer mental health, decreased quality of life, increased carer burden and social isolation.^5^, ^6^ This prevents timely diagnosis and therapeutic care.^7^ Therefore, dementia can have serious physical and psychosocial impacts on people living with it, their care partners and families.^8^, ^9^, ^10^

People living with dementia from culturally and linguistically diverse (CALD) backgrounds experience stigma at a heightened rate and are often shamed within the community.^11^, ^12^ Myriad beliefs can contribute to these sentiments, including not conceptualising dementia as a serious illness; considering dementia as a normal part of ageing; believing that dementia has spiritual, psychological or social causes; caring for someone with dementia as a family or personal obligation; believing nothing can be done to help their dementia and having a negative outlook due to negative healthcare experiences.^13^, ^14^, ^15^ While many organisations support people of CALD backgrounds who live with dementia, further efforts are required. For instance, Dementia Australia,^16^ Alzheimer's Society in the United Kingdom^17^ and the Alzheimer Society of Canada^18^ all offer online resources translated into various community languages. While helpful, these resources assume that recipients are: literate in their community language; as well as digitally literate. These (and perhaps other assumptions) can hinder access to information and support.^12^, ^19^, ^20^, ^21^, ^22^, ^23^, ^24^

Using bilingual workers and/or community leaders promotes the delivery of information in culturally appropriate and impactful ways, particularly when translating dementia into other languages.^25^ Clear and effective communication is key given that terms surrounding dementia can be stigmatising in other languages. For example, in Arabic, the term for dementia, ‘kharaf’, translates to ‘unravelled’ or ‘loss of mind’, while Vietnamese communities describe it as ‘lân lon’ or ‘mixed‐up’ and ‘gia lân’ or ‘old and confused’.^14^ Both cultures attribute dementia to normal ageing, psychological or spiritual/religious causes—these attributions can lead to misperceptions that are difficult to overcome due to low health literacy levels and limited understanding of medical terminology in CALD groups.^14^, ^26^, ^27^ Effective communication about dementia requires a culturally sensitive approach to overcome stigma and literacy barriers.^20^

The World Health Organisation's global action plan and the global dementia‐friendly communities initiative promote awareness of dementia through inclusion, enablement and empowerment of people with dementia and their care partners.^28^, ^29^ To implement these at a local level, organisations might refer to toolkits published by peak bodies (such as Dementia Australia's Dementia Friendly Communities toolkit)^30^ and/or the global Dementia Friends initiative.^31^ Originating in Japan, Dementia Friends is a community‐led education programme for the public to learn about disease‐focused information and socially supportive actions to promote inclusivity for people with dementia and their care partners.^32^, ^33^ Dementia Friends sessions have improved dementia knowledge and comfort levels with people with dementia in the United Kingdom and the United States of America.^34^, ^35^ The Dementia Friendly Kiama project in Australia involved people with dementia as spokespeople and resulted in more positive beliefs about dementia and awareness of local services.^36^, ^37^ However, the stigma faced by CALD people living with dementia might prevent them from serving in this capacity; thus, alternative advocacy options are necessary.

The Dementia Friends programme is typically designed for people who speak the main language of the host city or country. Multilingual or culturally inclusive dementia awareness‐raising programmes are lacking in Australian dementia‐friendly communities and across the world.^38^ In response to this gap, an online multilingual dementia information session was co‐created by the Canterbury Bankstown Dementia Alliance, comprising representatives from the Bankstown Dementia Carers Group, Western Sydney University, Canterbury‐Bankstown Council and multicultural service providers. The session was designed for use with English, Arabic and Vietnamese speaking individuals, as these were the three most spoken languages within Canterbury‐Bankstown where this study was set.^39^ The aims of this study were to: (1) characterise participants' perspectives about dementia; (2) examine the impact of this co‐created multilingual dementia information session and (3) identify factors that helped and hindered its implementation.

## 2 MATERIALS AND METHODS

### 2.1 Study design

This case study used multimethods research to achieve its aims. In stage one, a convergent parallel mixed methods approach^40^ was used to characterise dementia perceptions and establish the impact of the co‐created online dementia information session that was delivered in English, Arabic and Vietnamese. A pre‐/postassessment of the impact on dementia knowledge was not feasible, nor acceptable, given the literacy and stigma‐related concerns expressed by the CALD community representatives in the Dementia Alliance. In stage 2, a qualitative exploration of the initiative's implementation helpers and hinderers was undertaken.

### 2.2 Co‐creation of the dementia awareness initiative

The Dementia Alliance recognised that this was the first dementia awareness initiative to be conducted in the region and took care of its design and implementation. Guided by Dementia Australia's Dementia Friendly Community Toolkit,^30^ the dementia awareness initiative was co‐created with Canterbury Bankstown Dementia Alliance members representing each cultural group through a series of online meetings that were coordinated by the lead researcher.

The first meeting involved sharing cultural sensitivities and communication tips, particularly around the language and flow of information to be used when discussing dementia,^23^ for example, when the word ‘dementia’ first appeared to participants, facilitators were to acknowledge the stigma behind the word, encourage participants to reflect on this and ask them to remain open to learning about the condition. The next series of meetings involved mapping educational priorities, reviewing existing Dementia Friends resources for ideas,^41^ selecting appropriate topics that aligned with the dementia alliance's priorities, and creating relevant evaluation metrics (e.g., attendance rates, knowledge quizzes, surveys). Co‐creators also expressed a desire for the lead researcher to develop the material given their professional and academic expertise. Accordingly, the lead researcher developed the initial presentation content and evaluation material and iteratively revised this following feedback at co‐creation meetings until it was unanimously endorsed. The final version of the presentation material was translated from English into Arabic and Vietnamese using native speakers. The translated materials were emailed to the bilingual Dementia Alliance members to review accuracy and clarity; suggestions for revisions were incorporated by the translators.

### 2.3 Implementation of dementia awareness initiative

**Figure 1:**
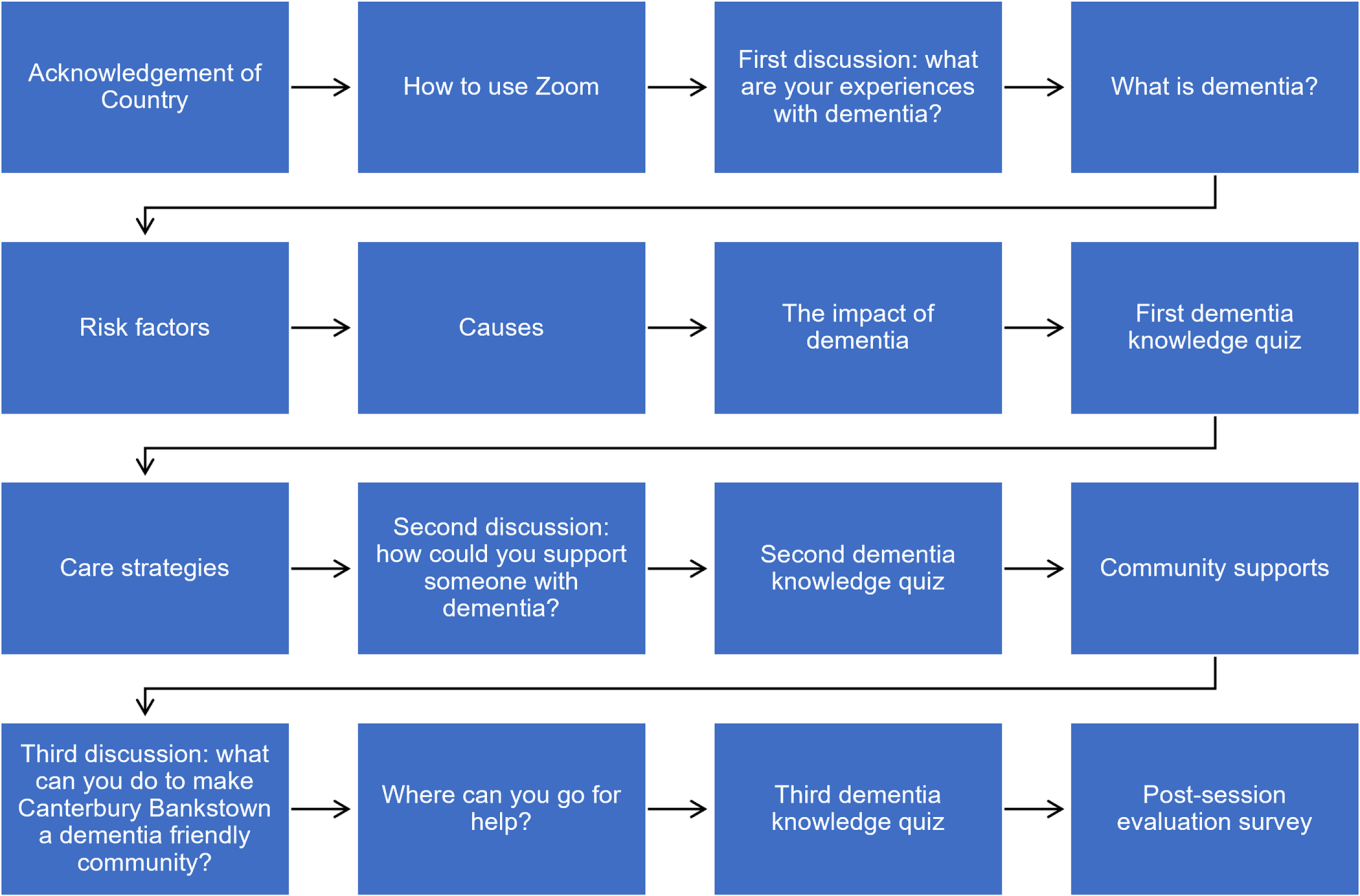
Sequence of the dementia information session.

The co‐created awareness initiative comprised an online 2‐h dementia information session delivered through a PowerPoint presentation, which included an Acknowledgement of the Country (to uphold Aboriginal and Torres Strait Islander cultural protocols), instructions to access the web‐conferencing platform—Zoom, a review of dementia, its risk factors, causes, impacts, care strategies, community supports and information on dementia‐related services. To facilitate engagement, three group discussions and three dementia knowledge quizzes were interspersed during the presentation (see Figure 1 for the information session's sequence of events).

The co‐convenor of the Canterbury Bankstown Dementia Alliance served as the Dementia Friends host for the English sessions and was present at the Arabic and Vietnamese sessions. Bilingual support workers were recruited through the multicultural service providers who are members of the Dementia Alliance to deliver the sessions in Arabic and Vietnamese. The bilingual facilitators completed training with the Dementia Friends host to ensure consistent delivery. The information sessions took place in September 2021, during Dementia Awareness month in Australia. Although face‐to‐face presentations are recommended for CALD communities,^20^ online delivery (using the Zoom platform) were necessary due to the COVID‐19 public health orders at that time.

### 2.4 Participants and recruitment

The sessions were promoted to English‐, Arabic‐ and Vietnamese‐speaking individuals, aged 18 years or over, living or working within Canterbury‐Bankstown, located in South‐Western Sydney, Australia. Language‐specific flyers were created and distributed via email and social media through the Canterbury Bankstown Dementia Alliance's members and associated networks for up to 6 weeks before each session. Digital promotion was used due to the aforesaid public health orders. Eventbrite was used to register participants, where an information sheet was provided, eligibility for the research was checked, and contact details were collected for the postsession survey. Consent to participate was implied through session registration. Participants were advised of this in the information sheet provided. The information sheet also emphasised that participation was entirely voluntary and could be discontinued at any time without giving reason. The study was approved by the Western Sydney University Human Research Ethics Committee (H14509).

Of the 114 people who registered (English *n* = 68; Arabic *n* = 13; Vietnamese *n* = 33), 87 participated in the sessions (English *n* = 48; Arabic *n* = 8; Vietnamese *n* = 33). Demographic details were not obtained during the registration process to protect participant privacy. This was necessary due to the mistrust in academic and governmental institutions that this multicultural community experienced during the COVID‐19 pandemic,^42^ as advised by Dementia Alliance members.

### 2.5 Evaluation approach

#### 2.5.1 Qualitative data collection and analysis

To qualitatively explore aims 1 and 2, data were collected from three open‐ended discussions facilitated by the facilitator during each information session. Participants were encouraged to respectfully reflect and respond to open‐ended questions. However, the facilitator did not push all participants to be involved to avoid discomfort or disengagement. The first discussion question, ‘what are your experiences with dementia?’, was asked at the start of the session to understand participants' baseline knowledge about dementia (aim 1). As the information session progressed, and participants were exposed to the material, the remaining group discussions were focused on the integration of knowledge (aim 2). The second discussion question, ‘how could you better support someone with dementia?’, was posed after participants received education about dementia causes, impacts and care strategies. The final question, ‘what can you do to make Canterbury‐Bankstown a dementia friendly community?’, was asked to prompt knowledge exchange regarding support strategies at the community level.

The facilitated discussions were not audio‐recorded due to the sensitive nature of the topics and the desire to maintain confidentiality. Instead, a bilingual researcher observed and summarised discussion points in the English and Arabic sessions. The Vietnamese note‐taker was unwell and unable to attend the session, which led to the absence of qualitative data for this language group. As we were focused on capturing discussion content, we did not track the number or level of engagement from participants. Notes documented during the group discussions were summarised in English for analysis in line with Hsieh and Shannon's conventional content analysis approach.^43^ Qualitative content analysis creates valid inferences from verbal, visual or written data, or any form of meaningful matter to describe and analyse specific phenomena.^43^ A bicultural Arabic‐Australian researcher repeatedly read the notes from the English and Arabic discussions each session to ensure understanding and search for meaning within and across the data. Data were coded according to the key concepts noted and codes were collated to create categories that reflected how different codes were related. These emergent categories were used to create meaningful clusters of data.

#### 2.5.2 Quantitative data collection and analysis

To supplement the qualitative group discussion data collected during the information sessions, and to further evaluate the initiative's impact (aim 2), two quantitative measures were developed with co‐creators: knowledge quizzes and a survey. This was necessary as co‐creators recommended using simple and brief evaluation tools (ideally those with ≤10 items), but no validated tool(s) were found to exist in the languages that were targeted here. Quiz and survey questions were selected based on a review of existing Dementia Friends resources, relevance to the Dementia Alliance's goals and select Alliance members' reporting obligations to employers/institutions.

**Figure 2:**
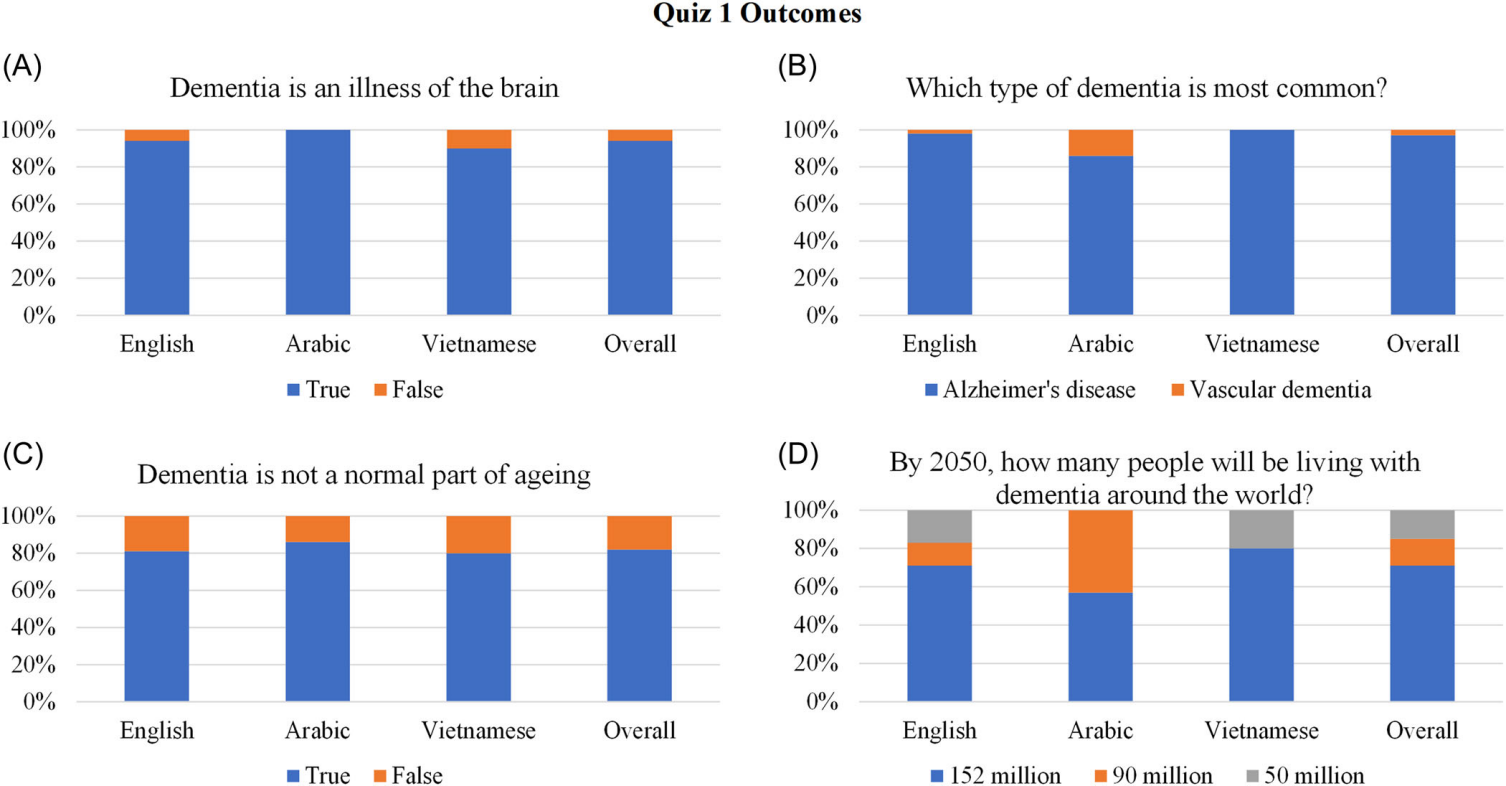
Quiz 1 items and outcomes concerning dementia knowledge (A–C) and prevalence (D). This figure demonstrates the percentage of participants who correctly responded to all four questions within Quiz 1. Percentages are given across the English, Arabic and Vietnamese speaking groups, as well as the overall percentage across the three language groups.

**Figure 3:**
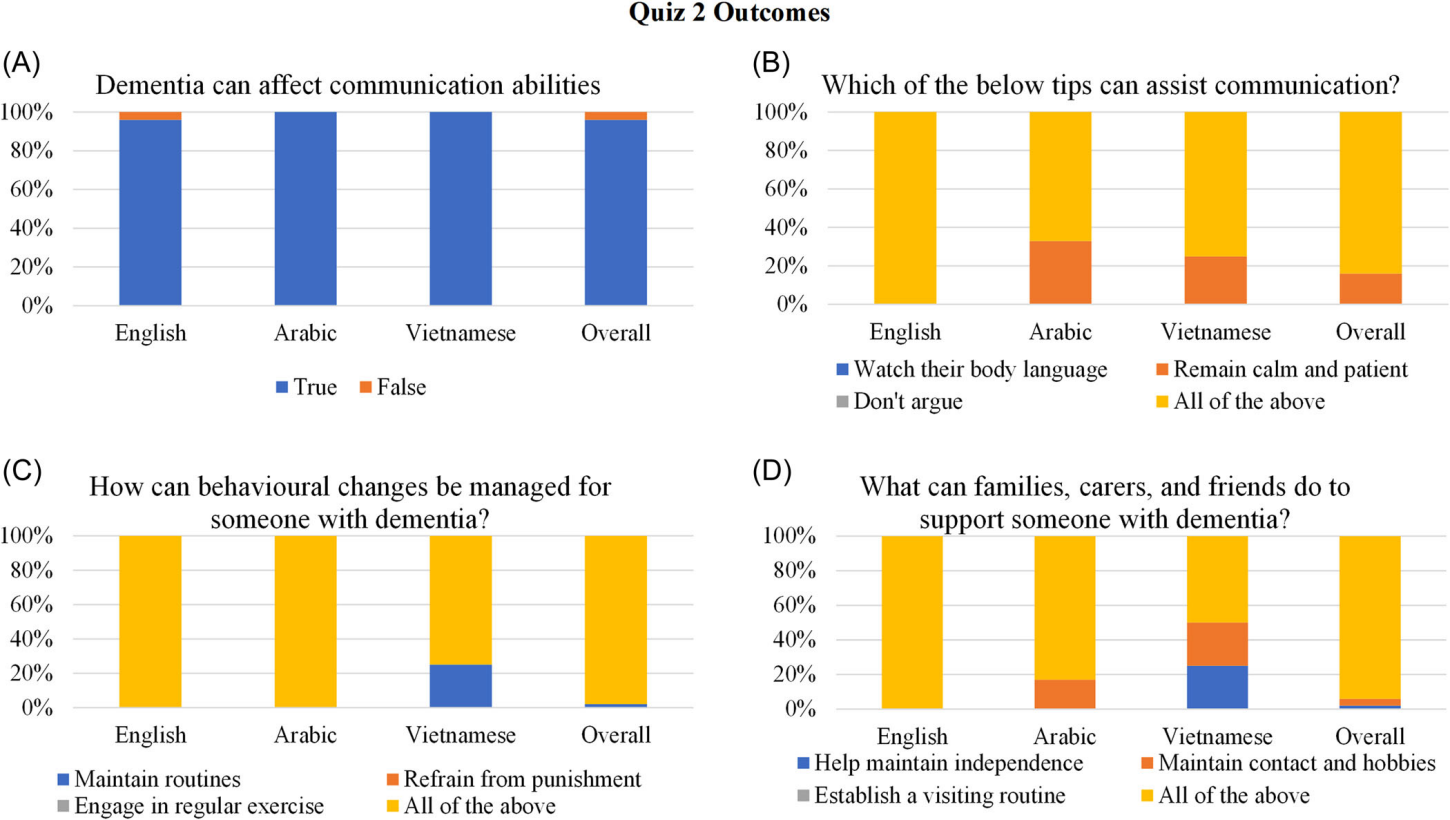
Quiz 2 outcomes regarding symptoms and management of dementia. This figure demonstrates the percentage of participants who correctly responded to all four questions within Quiz 2. Percentages are given across the English‐, Arabic‐ and Vietnamese‐speaking groups, as well as the overall percentage across the three language groups.

**Figure 4:**
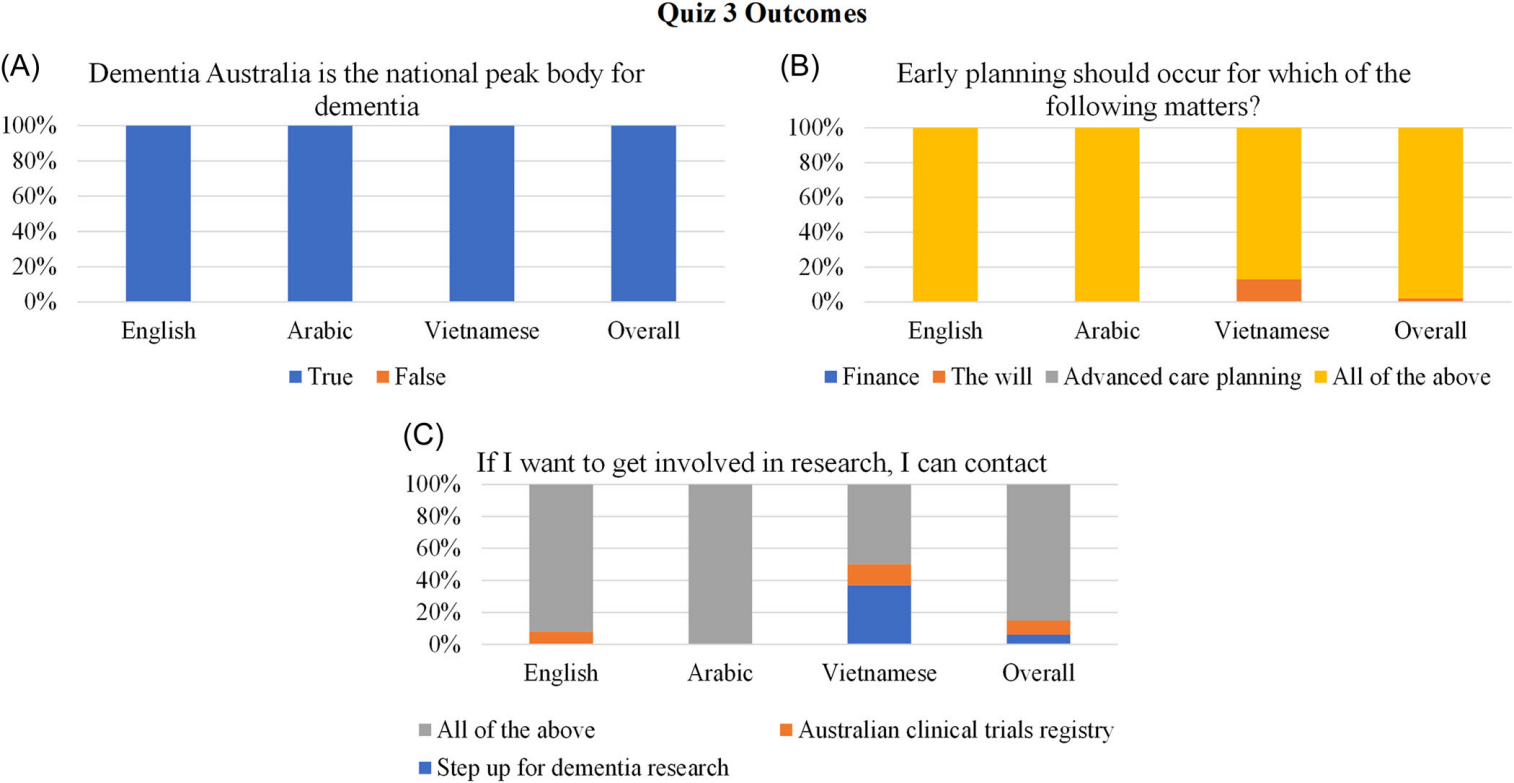
Quiz 3 outcomes regarding services for dementia.

Three quizzes were developed and administered throughout the information session, via Zoom's poll function, to assess knowledge learned. The first quiz had four questions probing dementia perceptions, types and prevalence; the second quiz contained four questions about symptoms and management and the third quiz had three items regarding knowledge about Australian dementia‐related services. A combination of multiple choice and true or false questions were used (see Figures 2, 3, 4 for the questions asked). The quizzes were administered using Zoom's polling function and results were downloaded into Microsoft Excel to compare performance across and between the three language groups.

**Figure 5:**
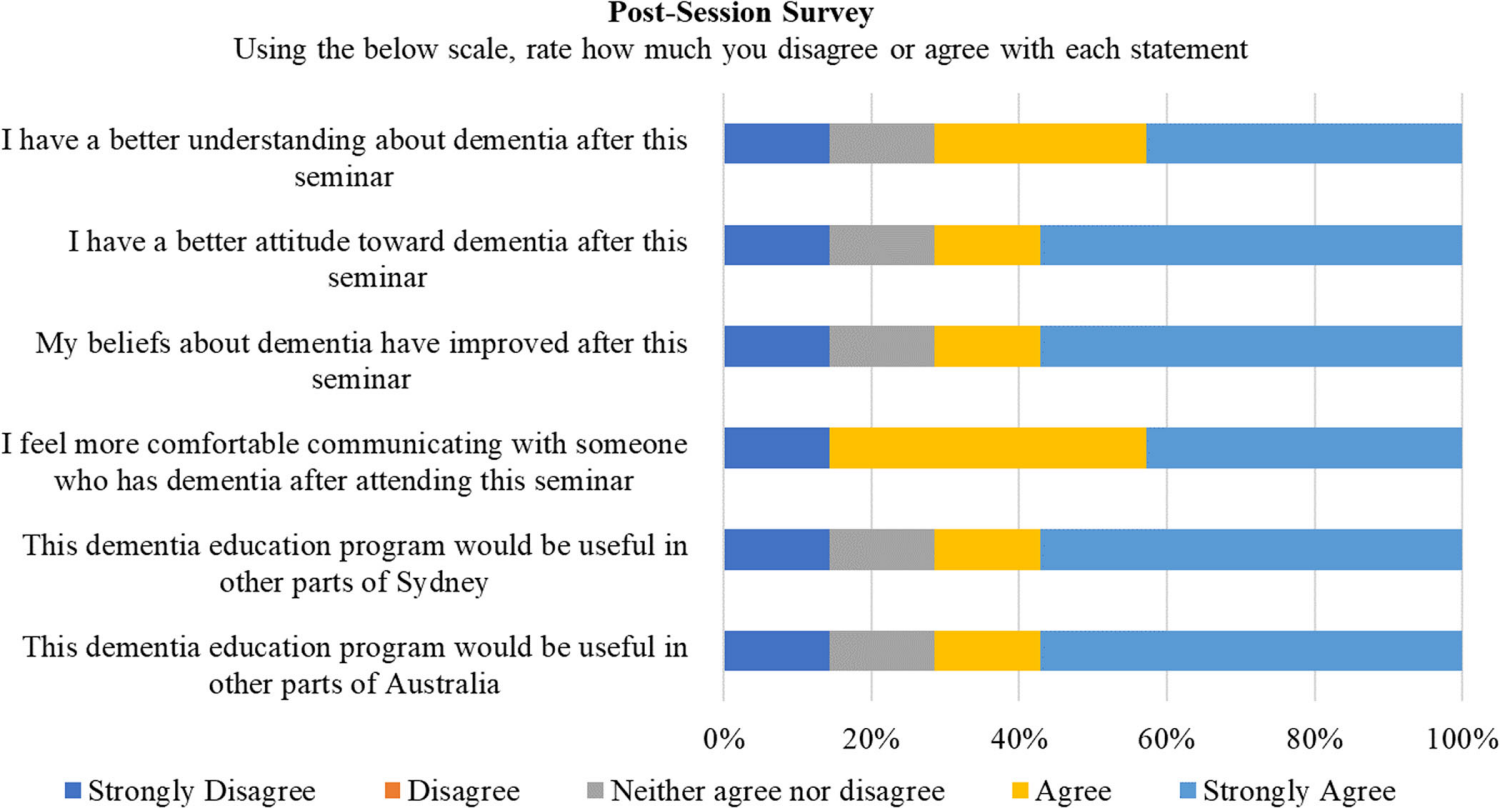
Postsession survey responses. This figure demonstrates the percentage of participant responses (*n* = 7) for each Likert scale item of the postsession survey.

Immediately after the information session, participants received a link to a six‐item postsession evaluation survey on the Qualtrics software programme. Respondents rated the extent of agreement or disagreement with statements regarding the impact of the session on their understanding, attitudes and beliefs about dementia, and their approaches towards people with dementia, on a 5‐point Likert scale. Statements also included whether the programme would be useful in other parts of Sydney and Australia (see Figure 5 for the questions asked). Demographic information, such as age, gender and occupation was also collected as part of this survey to help characterise the survey sample. The survey was only available in the English language due to resourcing limitations. Consequently, only 12 participants attempted the survey, and 7 participants fully completed it.

As this was a pilot study and exploratory in nature, statistical analyses were not conducted. Instead, descriptive analyses were undertaken on the data from the in‐session quizzes and postsession evaluation survey. Quiz performance was assessed across and between the three languages while postseminar survey responses were assessed across the seven participants. Findings were narratively synthesised to compare trends across and between the three languages.

The qualitative and quantitative findings were collated into a four‐page plain language report prepared by the research team and discussed with the Canterbury Bankstown Dementia Alliance at one of their quarterly meetings to aid interpretation. The report was emailed to all alliance members 2 weeks before the meeting to allow time for independent review and the meeting minutes documented their feedback for the lead researcher. The mixed‐methods findings were integrated into the discussion to address the first two aims of this study.

#### 2.5.3 Postimplementation evaluation of the dementia awareness initiative

A semistructured focus group was facilitated to understand the factors that helped or hindered the initiative's implementation (aim 3). This was conducted via Zoom with five members of the Canterbury Bankstown Dementia Alliance who were involved in the education initiative. Participants were invited to discuss: how the dementia alliance formed; what helped or hindered the formation of the alliance; why dementia education was deemed important; how the dementia alliance and the dementia education initiative were promoted; the factors that helped or hindered the initiative; strategies used to manage the barriers; how participant feedback was sourced and used and how momentum was sustained.

Following verbal consent, the focus group was digitally recorded. The digital recording was transcribed verbatim, and identifiable data were anonymised. The transcripts were checked and read repeatedly by a researcher before performing conventional content analysis on the collected data (as previously described).^43^ Findings were narratively synthesised.

## 3 RESULTS

The results from this multimethod study are reported as follows: (1) qualitative content analysis of notes from the English and Arabic language group discussions (noting that data were not collected for the Vietnamese group here); (2) quantitative outcomes from the knowledge quiz and postsession survey outcomes and (3) the postimplementation focus group exploring what helped or hindered the initiative.

### 3.1 Qualitative analysis outcomes

#### 3.1.1 Dementia perceptions and experiences

A few participants spoke from the perspective of having only professional experience as carers in the community who either worked with people living with dementia or worked in healthcare and were interested in learning more about this topic. Most participants shared that they had personal experience with dementia because one or more family members, mainly parents, had been diagnosed or it was suspected that they had dementia. Their family members with dementia ranged from only recently being diagnosed to being in the late stages and some had dementia‐related comorbidities.

#### 3.1.2 Arabic perspectives of dementia care

Several participants explained that they had not known much about dementia and needed to increase their awareness and understanding of themselves and others in their culture. They described the impact of stigma around dementia that made it hard to discuss or broach the subject with others. Because they associated dementia with mental illness, rather than a physical disease, this stigma was one reason why people might resist help. It was recognised that normalising the language around dementia was important to reduce stigma in the Arabic culture.

An Arabic participant explained that care was enacted by the family unit in her case, rather than being a role assigned to just one person. Participants described that it was important to shield their family members from the difficulties they experienced in caring for them. They spoke about the need to be patient and supportive.

Some Arabic participants explained that they found it difficult to navigate the health system in Australia. They were unsure where they could get support for carers in their local area. This was attributed to limited English and refugee status, which made paperwork and applying for support much more difficult. One participant was a bilingual carer who worked with people with dementia in the Arabic community and often provided resources and care to those clients; so, they regularly tried to increase awareness and connect people to resources and services as needed. However, it was discussed that more bilingual carers and workers experienced with the Arabic culture were needed. Additionally, dementia resources in Arabic and written in plain language were still needed and not widely accessible.

Also important to participants was increasing communication in the community and the number of home visits and support available. Several participants discussed the importance of education on dementia types, signs and risk reduction—they also emphasised the need to share this information with the broader community to reduce stigma and ensure people knew where to go for support. It was suggested that specific education around the importance of being patient, and not getting upset by someone asking too many questions or behaving differently in public would be helpful.

#### 3.1.3 Supporting people living with dementia

After receiving information about dementia causes, impact and care, participants from the English and Arabic groups discussed ways to support people living with dementia. These included keeping people comfortable, listening, communicating clearly, providing for their basic needs and quality of life, as well as being kind, gentle and patient. These types of support would help people to maintain their independence. Other ways of facilitating independence included letting them make decisions and letting them take their time in activities, rather than getting frustrated and taking over. Sharing this type of information would also help different carers, especially in large families, to know about what the person with dementia needed and preferred.

#### 3.1.4 Community inclusion of people living with dementia and carers

The final discussion was focused on the enablement of people living with dementia and their carers to participate in the community actively and meaningfully. According to the participants, this could involve places that people with dementia could visit with help from carers, dementia‐friendly activities in community spaces, increasing the accessibility of local places, including mobility considerations, and improving signage. Quiet and relaxing spaces for carers should be incorporated into shopping centres, much like it is for parents. Spaces that enabled carers to talk to other carers with access to informative resources might contribute to feelings of support, reducing loneliness and confusion. Carer wellbeing shopping hours might enable carers to shop, meet others and have accessible resources. These service designs reflect ways the community can care for carers by supporting their mental, physical and emotional health.

#### 3.1.5 Quantitative analysis outcomes

*Knowledge Quiz* 1: The first quiz assessed participants' general knowledge about dementia through four questions (see Figure 2). Here, 65 participants completed Quiz 1 (English *n* = 48, Arabic *n* = 7, Vietnamese *n* = 10). Nearly all participants correctly identified dementia as a brain illness, Alzheimer's disease as the most common type of dementia and there was little variation between the language groups regarding this (Figure 2A,B). Up to 20% of the English‐ and Vietnamese‐speaking groups, however, identified dementia as a normal part of ageing (Figure 2C). There was little variation between the English‐ and Vietnamese‐speaking groups in response to future dementia prevalence estimates; however, only 57% of the Arabic‐speaking group identified the correct estimated figure (Figure 2D).

*Knowledge Quiz* 2: The second quiz consisted of four questions regarding dementia symptoms (refer to Figure 3 for outcomes). Here, 54 participants completed it (English *n* = 44, Arabic *n* = 6, Vietnamese *n* = 4). Nearly all participants correctly recognised that dementia can affect communication abilities, with little variation between the language groups (Figure 3A). However, up to a third of the Arabic and Vietnamese participants did not correctly identify that watching body language, remaining calm and patient, and not arguing can assist in communication (Figure 3B). Most participants correctly recognised that maintaining routines, refraining from punishment and engaging in regular exercise can help to manage behavioural changes with dementia, with 25% of the Vietnamese participants responding incorrectly to this (Figure 3C). While most English and Arabic speaking participants identified that retaining independence, maintaining contact and hobbies, and establishing a visiting routine can support someone with dementia, this was not evidenced in half of the Vietnamese participants (Figure 3D).

*Knowledge Quiz* 3: The third quiz assessed knowledge about help services for those with dementia through three questions (refer to Figure 4). Here, 48 participants completed this quiz (English *n* = 37, Arabic *n* = 3, Vietnamese *n* = 8). All participants correctly identified Dementia Australia as the national peak body in Australia (Figure 4A) and 98% of participants understood that early planning should occur for finance, the will and advanced care planning (Figure 4B). Most English and Arabic speaking participants correctly identified Step Up for Dementia Research and the Australian Clinical Trials Registry for research engagement; however, this was only evidenced in half of the Vietnamese‐speaking participants.

#### 3.1.6 Postsession survey outcomes

Outlined below are the seven participant responses to the postseminar survey (see supplementary material for participant demographics). The participants were mostly female (*n* = 5), and all knew of a person living with dementia. Only one participant provided care or support to a person living with dementia. Figure 5 shows the survey responses where the agree and strongly agree responses, and the disagree and strongly disagree responses, were combined for easy reporting. Of the seven participants, 71% agreed that they had: a better understanding about dementia; an improved attitude towards dementia and improved beliefs about dementia after the session. Most reported: feeling more comfortable communicating with someone who has dementia after attending the session (86%); and that the dementia information session would be useful in other parts of Sydney and other parts of Australia (71%).

#### 3.1.7 Postimplementation focus group insights

Reflecting on how the initiative was implemented, members of the Canterbury Bankstown Dementia Alliance indicated that several factors helped or hindered it. The former included the support of an established organisation and feedback mechanisms; while the latter included the competing priorities and its limited accessibility to the target audiences. Each is addressed in turn.

The initiative was aided by the support of the local council—an established, local organisation with credibility and extensive networks. The council's standing served to raise the profile of the initiative as well as its perceived value:

[The council member] had the connections already established within the community. So, your older adults, some people with dementia, some families who have people affected by dementia, and the service providers … her role in the council enables her to do that. (member 1)

That's how we started. We didn't know whether we could do it. It was through council. (member 4)

Feedback mechanisms were also helpful. Members of the Canterbury Bankstown Dementia Alliance actively sought and were informed by feedback to improve their efforts to ultimately ensure the initiative offered culturally appropriate dementia information. Specifically, they invited participant comments through evaluative methods to formally appraise the initiative. They also engaged in regular reflection to informally deliberate on opportunities for improvement. According to the Alliance members, these feedback mechanisms bolstered their collective efforts:

With this project, they were very happy and very proud of the work, then I sent [the preliminary report] to everyone [at Council], and we just had very positive feedback … Those numbers are what's going to give us the evidence that these bureaucrats need to support us. (member 5)

We had our … meeting … where we broke down the report, we debriefed, we put in pointers about what needs to improve … we're that organised now … we said, we need to do this every year, not just this one time. (member 1)

Conversely, the initiative was hindered when other matters, including the COVID‐19 global pandemic, took precedence and the initiative was deemed relatively unimportant. When other priorities prevailed, there was limited time to dedicate to the initiative. This, in turn, compromised the ability of Alliance members to network widely and promote the initiative to bolster support:

I remember it taking a lot longer than expected … this was affected by COVID and the fact that we couldn't meet and had other priorities at the time … we tried to connect with the Dementia Friendly Kiama Group … we just got too busy to organise times. (member 1)

We're having a bit of difficulty because we are now working in partnership with another larger organisation … they don't really have the time to focus. (member 4)

The importance of prioritising the initiative was affirmed by the role of champions who were committed to the cause. According to Alliance members, the initiative prospered when they dedicated time and energy to it. This was typically because their lived experience enabled them to recognise its value for people living with dementia and their family members:

I am and have been very passionate about advocating for people with dementia, ever since my mum had it … you can see the difference that support makes to the families and to the person with dementia. (member 4)

I have 50% chance to develop dementia in the future. This is why I have a big passion about it. (member 5)

The initiative was also hindered when accessibility for the target audiences was limited by cultural insensitivity. Alliance members noted that the different multicultural communities were disengaged when the content and mode of delivery were not tailored to their needs and preferences. This included the need to translate the content into the appropriate and preferred language, given that dementia was often associated with stigma within these communities. Furthermore, while an online mode of delivery might offer an economical way to extend the reach of the initiative, it was often inaccessible to multicultural communities due to limited digital literacy:

In specific topics that are … hard for the community [to] grasp and that we're trying to get more engagement, it's better to actually have that expert in their own mother tongue … they also understand that cultural component of, and if it is a person that is trusted by that community, then they're gonna [sic] listen. (member 5)

In Arabic sessions … we have stopped using that word [kharaf] and are instead just saying Alzheimer's disease or in English, dementia. (member 1)

There is still long way to get people into familiar with online Webinar or training especially Vietnamese seniors. However, we can see this is the first step to connect them together through online for the information. (member 3)

## 4 DISCUSSION

Our findings indicate that an understanding of dementia was supported by the online multilingual dementia information sessions. As evidenced by the discussions, participants were personally connected to someone living with dementia and appreciated the need to increase education, adopt appropriate care strategies and increase the accessibility of resources and services. Discussions also confirmed the limited awareness of the illness and its association with stigma and discrimination.^5^, ^12^, ^14^ This was especially evident in the Arabic group where individuals found it difficult to discuss or broach the subject of dementia with others and advocated for normalising the language to reduce stigma—a recurring theme in CALD populations.^13^, ^14^, ^20^, ^44^ As the information session progressed and participants' knowledge was quizzed, performance metrics indicated that most participants understood information pertaining to dementia causes, types and impacts. However, the proportion of people responding incorrectly to quiz items suggests that information had not been retained. Ongoing dementia education and awareness campaigns are needed in communities to overcome misperceptions and promote help‐seeking.^22^ These recommendations extend beyond the CALD populations in Australia, given the overlapping information needs faced by immigrant or refugee populations in other parts of the world.^11^, ^13^, ^27^, ^38^, ^44^

Group discussions emphasised the importance of learning about and delivering appropriate care to people with dementia. Being aware of and meeting basic needs is crucial to person‐centred care.^45^, ^46^, ^47^, ^48^ Notably, the Arabic and Vietnamese participants tended to have poorer quiz performance regarding communication and care‐related questions. Considering this was the first time these communities were exposed to such information, there might have been challenges in processing and/or retaining knowledge. Information accessibility was recognised as a barrier in this initiative; this reinforces the importance of ongoing or perhaps more personalised approaches to address the knowledge gaps among CALD people.^13^, ^22^, ^44^

Addressing community accessibility and inclusion, as our information session did, fostered knowledge exchange about participant issues and needs that might not otherwise have been expressed or reported. Discussions confirmed that social and community engagement among people with dementia is severely impacted. While the Arabic group attributed this to stigma and fear of judgement, the English group recognised this because of the built environment's accessibility. Reflecting previous research,^49^ the English group highlighted that local areas were not dementia‐friendly, deterring people with dementia from participating in the community; as such, they called for more dementia‐inclusive social activities.

Barriers to accessing relevant support were highlighted, necessitating information about such services in education initiatives.^50^ The Arabic group attributed this difficulty to language barriers and limited culture‐specific services and information, echoing prior reports concerning CALD people.^12^, ^19^, ^20^, ^21^, ^22^, ^44^ The English group also struggled with locating the information within their community and advocated for better resources for care partners; but this consideration was not present among the Arabic group. Although some researchers have criticised the emphasis on care‐partners,^51^ the English group shared sentiments regarding the dismissal of the work care‐partners do and how this negatively affected their health. They recommended that services find a balance between prioritising the person with dementia and their care partner to deliver mutually beneficial support, as we have observed elsewhere.^52^ Understanding these barriers, at a local level, is crucial for providing accessible and relevant aid—a core component of dementia‐friendly communities or dementia‐inclusive societies. Notably, there is limited work being done with CALD or minoritised populations in dementia‐friendly initiatives across the world.^38^ We therefore encourage researchers, organisations and advocacy groups to collaborate with these groups from the outset of any community initiative or intervention, as was done here.^53^

The postseminar survey showed that the information session enhanced understandings, attitudes and beliefs about dementia, and that participants were more comfortable communicating with someone who has dementia.^34^, ^35^, ^36^, ^37^ These findings are, however, limited to a small proportion of participants and cannot be generalised, particularly as they pertain to individuals who can read and respond to questions in the English language. There were several other logistical challenges that prevented complete engagement with non‐English speaking participants. For example, discussion responses were not collected for the Vietnamese group, limiting the qualitative data analysed. Researchers must be adequately resourced to ensure inclusivity throughout the entirety of the programme. To maintain confidence with participants, we did not audio‐record group discussions and this limited the qualitative data presented here. This presents another barrier to research with underrepresented populations that needs careful consideration. We need to recognise alternative data collection tools and techniques when working with these cohorts, such as ensuring a fluent scribe is available to support notetaking that includes verbatim quotes.^54^ Furthermore, quiz engagement appeared to decrease as the information session progressed, particularly in the Arabic and Vietnamese groups. This may reflect a decline in participants' interest and attention due to the session's duration and/or relevance of the material. Future studies could explore these aspects of an initiative's engagement and design through follow‐up interviews or survey questions. Such initiatives would also benefit from longer‐term evaluations and, where possible, pre‐/postassessments of participants' dementia knowledge, beliefs and/or attitudes.^36^ The limitations to resourcing and online delivery mode echo the barriers faced by other researchers and community groups when conducting culturally inclusive dementia‐friendly initiatives.^38^

## 5 CONCLUSION

As demonstrated in the qualitative evaluation of the implementation, this initiative did well to leverage existing entities and networks to raise awareness using a community‐minded approach to sustain action through feedback mechanisms. These facilitators align with previous recommendations for dementia information provision in CALD communities thus serving as a practical guide for other researchers and/or advocacy bodies.^15^, ^20^, ^30^, ^44^ Co‐creation is a necessary component of dementia‐friendly initiatives, enabling stakeholders to address unmet educational needs in multicultural communities.^55^ Information should also be translated in a sensitive manner to ensure appropriate terminology and information flow when discussing dementia.^15^, ^20^ This was one of the first dementia awareness initiatives run as part of a dementia‐friendly community in a multicultural region that has not been exposed to such information before. The sessions supported participants' understandings of causes, impacts, and care, as well as highlighted the challenges associated with accessing resources and support. Although our findings are based on a small sample, they demonstrate the importance of delivering dementia education to CALD communities to improve how people living with the illness are perceived and treated.

## AUTHOR CONTRIBUTIONS

**Yousra Ali**: Formal analysis; data curation; investigation; visualisation; writing—original draft; software. **Gabriela E. Caballero**: investigation; writing—review and editing; resources; project administration. **Eman Shatnawi**: Methodology; software; data curation; validation; investigation; funding acquisition; writing—original draft; project administration; resources. **Ann Dadich**: Conceptualisation; methodology; data curation; formal analysis; validation; funding acquisition; project administration; resources; writing—review and editing; writing—original draft; supervision. **Canterbury Bankstown Dementia Alliance**: Resources; project administration; funding acquisition; writing—original draft; writing—review and editing; conceptualisation; methodology; data curation. **Genevieve Z. Steiner‐Lim**: Conceptualisation; methodology; writing— original draft; writing—review and editing; funding acquisition; project administration; resources; supervision. **Michelle DiGiacomo**: Conceptualisation; methodology; formal analysis; validation; investigation; funding acquisition; writing—original draft; writing— review and editing; project administration; resources; supervision. **Diana Karamacoska**: Conceptualisation; methodology; software; data curation; supervision; formal analysis; validation; investigation; funding acquisition; writing—original draft; writing—review and editing; visualisation; project administration; resources.

## CONFLICT OF INTEREST STATEMENT

As a medical research institute, NICM receives research grants and donations from foundations, universities, government agencies, individuals and industry. Sponsors and donors provide untied funding for work to advance the vision and mission of the Institute. The project that is the subject of this article was not undertaken as part of a contractual relationship with any organisation other than the funding declared. Genevieve Z. Steiner‐Lim and Diana Karamacoska are employed by NICM and are members of the Canterbury‐Bankstown Alliance. The remaining authors declare no conflict of interest.

## ETHICS STATEMENT

The study was approved by the Western Sydney University Human Research Ethics Committee (reference number: H14509).

## Supporting information

Supporting information.

## Data Availability

The participants of this study did not give written consent for their data to be shared publicly, so due to the sensitive nature of the research, supporting data is not available.
