# Supplementary material for: Assessing the impact of an online dementia awareness initiative co‐created with and for English, Arabic and Vietnamese speaking communities: A case study
Source: Health Expect. 2024 Apr 15;27(2):e14026. doi: 10.1111/hex.14026 (PMC11017301; doi:10.1111/hex.14026)
Supplement: Supplementary file 1 — Supporting information. [file HEX-27-e14026-s001.docx]

# Supplementary Material

| **Table S1**. Demographics of post-seminar survey participants | |
| --- | --- |
| **Demographic Information** | **Number of Participants** |
| Total respondents  Complete surveys | 12  7 |
| Age range, years (average ± SD) | 30–72 (53 ± 17.5) |
| **Gender** | |
| Female | 5 |
| Male | 2 |
| **Place of birth** | |
| Australia | 3 |
| Asia | 2 |
| Middle East | 1 |
| Europe | 1 |
| **Languages spoken (other than English)** |  |
| English  Arabic | 4  2 |
| Vietnamese | 1 |
| **Occupation** |  |
| Unemployed/not working | 3 |
| Education and training | 1 |
| Healthcare/social assistance | 3 |
| **Education** |  |
| High school diploma | 3 |
| University degree  Other | 3  1 |
| **Salary**  Below $25,000  $25,000 - $40,000  $40,001 - $70,000  $70,001 or more  No response  **Relationship with dementia** | 1  3  1  1  1 |
| Know a person living with dementia | 7 |
| Provide care/support for a person living with dementia | 1 |
| **Aware of DFCB project**  No  Yes  **If yes, how?**  Dementia education seminar | 2  5  5 |

*Note. DFCB = Dementia Friendly Canterbury-Bankstown*
